# Supplementary material for: Pyruvate Dehydrogenase Kinase 1 inhibition mediated oxidative phosphorylation enhancement in cartilage promotes osteoarthritis progression
Source: BMC Musculoskelet Disord. 2023 Jul 20;24:597. doi: 10.1186/s12891-023-06585-6 (PMC10357736; doi:10.1186/s12891-023-06585-6)
Supplement: Supplementary file 1 — Additional file 1. [file 12891_2023_6585_MOESM1_ESM.zip › BP.Gsea.1653623667859/GOBP_REGULATION_OF_MRNA_SPLICING_VIA_SPLICEOSOME.html]

Details for gene set GOBP\_REGULATION\_OF\_MRNA\_SPLICING\_VIA\_SPLICEOSOME[GSEA]

|  || Dataset | 55457\_collapsed\_to\_symbols.cls.cls #OA\_versus\_normal.cls.cls #OA\_versus\_normal\_repos |
| Phenotype | cls.cls#OA\_versus\_normal\_repos |
| Upregulated in class | normal |
| GeneSet | GOBP\_REGULATION\_OF\_MRNA\_SPLICING\_VIA\_SPLICEOSOME |
| Enrichment Score (ES) | -0.50560445 |
| Normalized Enrichment Score (NES) | -2.1741483 |
| Nominal p-value | 0.0 |
| FDR q-value | 0.0036141332 |
| FWER p-Value | 0.018 |
Table: GSEA Results Summary

  

Fig 1: Enrichment plot: GOBP\_REGULATION\_OF\_MRNA\_SPLICING\_VIA\_SPLICEOSOME      
 Profile of the Running ES Score & Positions of GeneSet Members on the Rank Ordered List

  

| SYMBOL | TITLE | RANK IN GENE LIST | RANK METRIC SCORE | RUNNING ES | CORE ENRICHMENT || 1 | FAM172A | family with sequence similarity 172 member A [Source:HGNC Symbol;Acc:HGNC:25365] | 251 | 0.785 | 0.0046 | No |
| 2 | SRSF9 | serine and arginine rich splicing factor 9 [Source:HGNC Symbol;Acc:HGNC:10791] | 882 | 0.548 | -0.0277 | No |
| 3 | SAP18 | Sin3A associated protein 18 [Source:HGNC Symbol;Acc:HGNC:10530] | 1099 | 0.496 | -0.0292 | No |
| 4 | MBNL3 | muscleblind like splicing regulator 3 [Source:HGNC Symbol;Acc:HGNC:20564] | 1165 | 0.483 | -0.0194 | No |
| 5 | RBM23 | RNA binding motif protein 23 [Source:HGNC Symbol;Acc:HGNC:20155] | 1678 | 0.402 | -0.0470 | No |
| 6 | RBM4 | RNA binding motif protein 4 [Source:HGNC Symbol;Acc:HGNC:9901] | 1970 | 0.364 | -0.0584 | No |
| 7 | RBM7 | RNA binding motif protein 7 [Source:HGNC Symbol;Acc:HGNC:9904] | 2620 | 0.288 | -0.1002 | No |
| 8 | RAVER2 | "ribonucleoprotein, PTB binding 2 [Source:HGNC Symbol;Acc:HGNC:25577]" | 2956 | 0.254 | -0.1185 | No |
| 9 | FXR1 | FMR1 autosomal homolog 1 [Source:HGNC Symbol;Acc:HGNC:4023] | 3012 | 0.249 | -0.1151 | No |
| 10 | C1QBP | complement C1q binding protein [Source:HGNC Symbol;Acc:HGNC:1243] | 3083 | 0.241 | -0.1131 | No |
| 11 | PUF60 | poly(U) binding splicing factor 60 [Source:HGNC Symbol;Acc:HGNC:17042] | 3237 | 0.227 | -0.1181 | No |
| 12 | SON | SON DNA and RNA binding protein [Source:HGNC Symbol;Acc:HGNC:11183] | 3374 | 0.214 | -0.1221 | No |
| 13 | LARP7 | "La ribonucleoprotein 7, transcriptional regulator [Source:HGNC Symbol;Acc:HGNC:24912]" | 3395 | 0.212 | -0.1171 | No |
| 14 | CIRBP | cold inducible RNA binding protein [Source:HGNC Symbol;Acc:HGNC:1982] | 3441 | 0.207 | -0.1142 | No |
| 15 | RBFOX2 | RNA binding fox-1 homolog 2 [Source:HGNC Symbol;Acc:HGNC:9906] | 3523 | 0.200 | -0.1144 | No |
| 16 | MBNL2 | muscleblind like splicing regulator 2 [Source:HGNC Symbol;Acc:HGNC:16746] | 3524 | 0.200 | -0.1082 | No |
| 17 | KHDRBS3 | "KH RNA binding domain containing, signal transduction associated 3 [Source:HGNC Symbol;Acc:HGNC:18117]" | 3714 | 0.184 | -0.1173 | No |
| 18 | KHDRBS1 | "KH RNA binding domain containing, signal transduction associated 1 [Source:HGNC Symbol;Acc:HGNC:18116]" | 3748 | 0.182 | -0.1143 | No |
| 19 | MAGOH | "mago homolog, exon junction complex subunit [Source:HGNC Symbol;Acc:HGNC:6815]" | 4266 | 0.131 | -0.1506 | No |
| 20 | KHDRBS2 | "KH RNA binding domain containing, signal transduction associated 2 [Source:HGNC Symbol;Acc:HGNC:18114]" | 4550 | 0.109 | -0.1693 | No |
| 21 | PCBP4 | poly(rC) binding protein 4 [Source:HGNC Symbol;Acc:HGNC:8652] | 4577 | 0.107 | -0.1681 | No |
| 22 | PTBP1 | polypyrimidine tract binding protein 1 [Source:HGNC Symbol;Acc:HGNC:9583] | 4650 | 0.100 | -0.1706 | No |
| 23 | SNW1 | SNW domain containing 1 [Source:HGNC Symbol;Acc:HGNC:16696] | 5472 | 0.035 | -0.2336 | No |
| 24 | RBM42 | RNA binding motif protein 42 [Source:HGNC Symbol;Acc:HGNC:28117] | 6059 | -0.009 | -0.2790 | No |
| 25 | RBFOX1 | RNA binding fox-1 homolog 1 [Source:HGNC Symbol;Acc:HGNC:18222] | 6152 | -0.016 | -0.2857 | No |
| 26 | WTAP | WT1 associated protein [Source:HGNC Symbol;Acc:HGNC:16846] | 6598 | -0.049 | -0.3189 | No |
| 27 | RBM10 | RNA binding motif protein 10 [Source:HGNC Symbol;Acc:HGNC:9896] | 6647 | -0.053 | -0.3210 | No |
| 28 | SF3B4 | splicing factor 3b subunit 4 [Source:HGNC Symbol;Acc:HGNC:10771] | 6777 | -0.061 | -0.3292 | No |
| 29 | PRDX6 | peroxiredoxin 6 [Source:HGNC Symbol;Acc:HGNC:16753] | 6783 | -0.061 | -0.3277 | No |
| 30 | RBM15B | RNA binding motif protein 15B [Source:HGNC Symbol;Acc:HGNC:24303] | 6867 | -0.069 | -0.3321 | No |
| 31 | SMU1 | SMU1 DNA replication regulator and spliceosomal factor [Source:HGNC Symbol;Acc:HGNC:18247] | 7299 | -0.102 | -0.3626 | No |
| 32 | NOVA1 | NOVA alternative splicing regulator 1 [Source:HGNC Symbol;Acc:HGNC:7886] | 7394 | -0.110 | -0.3665 | No |
| 33 | DYRK1A | dual specificity tyrosine phosphorylation regulated kinase 1A [Source:HGNC Symbol;Acc:HGNC:3091] | 7667 | -0.132 | -0.3837 | No |
| 34 | MYOD1 | myogenic differentiation 1 [Source:HGNC Symbol;Acc:HGNC:7611] | 7839 | -0.143 | -0.3926 | No |
| 35 | FMR1 | FMRP translational regulator 1 [Source:HGNC Symbol;Acc:HGNC:3775] | 7944 | -0.151 | -0.3960 | No |
| 36 | RNPS1 | RNA binding protein with serine rich domain 1 [Source:HGNC Symbol;Acc:HGNC:10080] | 8275 | -0.177 | -0.4163 | No |
| 37 | HNRNPA2B1 | heterogeneous nuclear ribonucleoprotein A2/B1 [Source:HGNC Symbol;Acc:HGNC:5033] | 8576 | -0.200 | -0.4336 | No |
| 38 | RBM8A | RNA binding motif protein 8A [Source:HGNC Symbol;Acc:HGNC:9905] | 8662 | -0.205 | -0.4339 | No |
| 39 | RBM3 | RNA binding motif protein 3 [Source:HGNC Symbol;Acc:HGNC:9900] | 8859 | -0.222 | -0.4424 | No |
| 40 | HNRNPL | heterogeneous nuclear ribonucleoprotein L [Source:HGNC Symbol;Acc:HGNC:5045] | 8892 | -0.224 | -0.4380 | No |
| 41 | RBMX | RNA binding motif protein X-linked [Source:HGNC Symbol;Acc:HGNC:9910] | 8938 | -0.227 | -0.4345 | No |
| 42 | RBMY1A1 | RNA binding motif protein Y-linked family 1 member A1 [Source:HGNC Symbol;Acc:HGNC:9912] | 9014 | -0.233 | -0.4331 | No |
| 43 | HSPA8 | heat shock protein family A (Hsp70) member 8 [Source:HGNC Symbol;Acc:HGNC:5241] | 9140 | -0.243 | -0.4354 | No |
| 44 | NCBP1 | nuclear cap binding protein subunit 1 [Source:HGNC Symbol;Acc:HGNC:7658] | 9149 | -0.244 | -0.4285 | No |
| 45 | TRA2B | transformer 2 beta homolog [Source:HGNC Symbol;Acc:HGNC:10781] | 9243 | -0.251 | -0.4280 | No |
| 46 | HNRNPU | heterogeneous nuclear ribonucleoprotein U [Source:HGNC Symbol;Acc:HGNC:5048] | 9847 | -0.306 | -0.4656 | No |
| 47 | SRSF3 | serine and arginine rich splicing factor 3 [Source:HGNC Symbol;Acc:HGNC:10785] | 10280 | -0.349 | -0.4886 | No |
| 48 | RBM15 | RNA binding motif protein 15 [Source:HGNC Symbol;Acc:HGNC:14959] | 10499 | -0.373 | -0.4941 | Yes |
| 49 | SRSF2 | serine and arginine rich splicing factor 2 [Source:HGNC Symbol;Acc:HGNC:10783] | 10507 | -0.374 | -0.4831 | Yes |
| 50 | SRPK2 | SRSF protein kinase 2 [Source:HGNC Symbol;Acc:HGNC:11306] | 10522 | -0.376 | -0.4726 | Yes |
| 51 | MBNL1 | muscleblind like splicing regulator 1 [Source:HGNC Symbol;Acc:HGNC:6923] | 10557 | -0.379 | -0.4636 | Yes |
| 52 | SRSF6 | serine and arginine rich splicing factor 6 [Source:HGNC Symbol;Acc:HGNC:10788] | 10659 | -0.389 | -0.4595 | Yes |
| 53 | YTHDC1 | YTH domain containing 1 [Source:HGNC Symbol;Acc:HGNC:30626] | 10687 | -0.391 | -0.4495 | Yes |
| 54 | RBM25 | RNA binding motif protein 25 [Source:HGNC Symbol;Acc:HGNC:23244] | 10800 | -0.405 | -0.4458 | Yes |
| 55 | CELF2 | CUGBP Elav-like family member 2 [Source:HGNC Symbol;Acc:HGNC:2550] | 10832 | -0.409 | -0.4356 | Yes |
| 56 | RBM5 | RNA binding motif protein 5 [Source:HGNC Symbol;Acc:HGNC:9902] | 10849 | -0.410 | -0.4242 | Yes |
| 57 | TIA1 | TIA1 cytotoxic granule associated RNA binding protein [Source:HGNC Symbol;Acc:HGNC:11802] | 10855 | -0.411 | -0.4119 | Yes |
| 58 | NUP98 | nucleoporin 98 and 96 precursor [Source:HGNC Symbol;Acc:HGNC:8068] | 10875 | -0.413 | -0.4006 | Yes |
| 59 | SRSF10 | serine and arginine rich splicing factor 10 [Source:HGNC Symbol;Acc:HGNC:16713] | 10926 | -0.422 | -0.3916 | Yes |
| 60 | SRSF7 | serine and arginine rich splicing factor 7 [Source:HGNC Symbol;Acc:HGNC:10789] | 10930 | -0.422 | -0.3788 | Yes |
| 61 | PRPF19 | pre-mRNA processing factor 19 [Source:HGNC Symbol;Acc:HGNC:17896] | 11269 | -0.467 | -0.3908 | Yes |
| 62 | SRRM1 | serine and arginine repetitive matrix 1 [Source:HGNC Symbol;Acc:HGNC:16638] | 11302 | -0.471 | -0.3787 | Yes |
| 63 | METTL16 | "methyltransferase 16, N6-methyladenosine [Source:HGNC Symbol;Acc:HGNC:28484]" | 11346 | -0.478 | -0.3673 | Yes |
| 64 | RBM39 | RNA binding motif protein 39 [Source:HGNC Symbol;Acc:HGNC:15923] | 11434 | -0.493 | -0.3589 | Yes |
| 65 | CELF3 | CUGBP Elav-like family member 3 [Source:HGNC Symbol;Acc:HGNC:11967] | 11510 | -0.502 | -0.3493 | Yes |
| 66 | ZBTB7A | zinc finger and BTB domain containing 7A [Source:HGNC Symbol;Acc:HGNC:18078] | 11600 | -0.516 | -0.3404 | Yes |
| 67 | SF1 | splicing factor 1 [Source:HGNC Symbol;Acc:HGNC:12950] | 11664 | -0.527 | -0.3290 | Yes |
| 68 | DDX17 | DEAD-box helicase 17 [Source:HGNC Symbol;Acc:HGNC:2740] | 11716 | -0.535 | -0.3165 | Yes |
| 69 | SRPK1 | SRSF protein kinase 1 [Source:HGNC Symbol;Acc:HGNC:11305] | 11889 | -0.569 | -0.3124 | Yes |
| 70 | NOVA2 | NOVA alternative splicing regulator 2 [Source:HGNC Symbol;Acc:HGNC:7887] | 12006 | -0.594 | -0.3031 | Yes |
| 71 | SNRNP70 | small nuclear ribonucleoprotein U1 subunit 70 [Source:HGNC Symbol;Acc:HGNC:11150] | 12073 | -0.613 | -0.2894 | Yes |
| 72 | FXR2 | FMR1 autosomal homolog 2 [Source:HGNC Symbol;Acc:HGNC:4024] | 12219 | -0.650 | -0.2806 | Yes |
| 73 | U2AF2 | U2 small nuclear RNA auxiliary factor 2 [Source:HGNC Symbol;Acc:HGNC:23156] | 12226 | -0.652 | -0.2610 | Yes |
| 74 | NCL | nucleolin [Source:HGNC Symbol;Acc:HGNC:7667] | 12255 | -0.662 | -0.2428 | Yes |
| 75 | QKI | "QKI, KH domain containing RNA binding [Source:HGNC Symbol;Acc:HGNC:21100]" | 12340 | -0.688 | -0.2281 | Yes |
| 76 | HNRNPK | heterogeneous nuclear ribonucleoprotein K [Source:HGNC Symbol;Acc:HGNC:5044] | 12341 | -0.688 | -0.2069 | Yes |
| 77 | HNRNPA1 | heterogeneous nuclear ribonucleoprotein A1 [Source:HGNC Symbol;Acc:HGNC:5031] | 12356 | -0.693 | -0.1866 | Yes |
| 78 | REST | RE1 silencing transcription factor [Source:HGNC Symbol;Acc:HGNC:9966] | 12382 | -0.703 | -0.1669 | Yes |
| 79 | CELF1 | CUGBP Elav-like family member 1 [Source:HGNC Symbol;Acc:HGNC:2549] | 12391 | -0.704 | -0.1458 | Yes |
| 80 | THRAP3 | thyroid hormone receptor associated protein 3 [Source:HGNC Symbol;Acc:HGNC:22964] | 12426 | -0.717 | -0.1264 | Yes |
| 81 | TRA2A | transformer 2 alpha homolog [Source:HGNC Symbol;Acc:HGNC:16645] | 12479 | -0.740 | -0.1076 | Yes |
| 82 | JMJD6 | "jumonji domain containing 6, arginine demethylase and lysine hydroxylase [Source:HGNC Symbol;Acc:HGNC:19355]" | 12658 | -0.832 | -0.0958 | Yes |
| 83 | DAZAP1 | DAZ associated protein 1 [Source:HGNC Symbol;Acc:HGNC:2683] | 12686 | -0.854 | -0.0716 | Yes |
| 84 | SFSWAP | splicing factor SWAP [Source:HGNC Symbol;Acc:HGNC:10790] | 12747 | -0.908 | -0.0483 | Yes |
| 85 | SRSF4 | serine and arginine rich splicing factor 4 [Source:HGNC Symbol;Acc:HGNC:10786] | 12749 | -0.911 | -0.0203 | Yes |
| 86 | DDX5 | DEAD-box helicase 5 [Source:HGNC Symbol;Acc:HGNC:2746] | 12834 | -1.038 | 0.0052 | Yes |
Table: GSEA details [plain text format]

  

Fig 2: GOBP\_REGULATION\_OF\_MRNA\_SPLICING\_VIA\_SPLICEOSOME      
 Blue-Pink O' Gram in the Space of the Analyzed GeneSet

  

Fig 3: GOBP\_REGULATION\_OF\_MRNA\_SPLICING\_VIA\_SPLICEOSOME: Random ES distribution      
 Gene set null distribution of ES for **GOBP\_REGULATION\_OF\_MRNA\_SPLICING\_VIA\_SPLICEOSOME**
